# Internet-Based Cognitive Behavioral Therapy for Residual Symptoms in Bipolar Disorder Type II: A Single-Subject Design Pilot Study

**DOI:** 10.2196/resprot.3910

**Published:** 2015-04-23

**Authors:** Fredrik Holländare, Annsofi Eriksson, Lisa Lövgren, Mats B Humble, Katja Boersma

**Affiliations:** ^1^Psychiatric Research CentreSchool of Health and Medical ScienceÖrebro UniversityÖrebroSweden; ^2^School of Law, Psychology and Social workÖrebro UniversityÖrebroSweden; ^3^Department of PsychiatryFaculty of Medicine and HealthÖrebro UniversityÖrebroSweden

**Keywords:** bipolar disorder, Internet, cognitive therapy, behavioral therapy, pilot projects

## Abstract

**Background:**

Bipolar disorder is a chronic condition with recurring episodes that often lead to suffering, decreased functioning, and sick leave. Pharmacotherapy in the form of mood stabilizers is widely available, but does not eliminate the risk of a new depressive or (hypo)manic episode. One way to reduce the risk of future episodes is to combine pharmacological treatment with individual or group psychological interventions. However, access to such interventions is often limited due to a shortage of trained therapists. In unipolar depression there is now robust evidence of the effectiveness of Internet-based psychological interventions, usually comprising psychoeducation and cognitive behavioral therapy (CBT). Internet-based interventions for persons suffering from bipolar disorder could increase access to psychological treatment.

**Objective:**

The aim of this study was to investigate the feasibility of an Internet-based intervention, as well as its effect on residual depressive symptoms in persons diagnosed with bipolar disorder type II (BP-II). The most important outcomes were depressive symptoms, treatment adherence, and whether the patient perceived the intervention as helpful.

**Methods:**

A total of 7 patients diagnosed with bipolar disorder type II at a Swedish psychiatric outpatient clinic were offered the opportunity to participate. Of the 7 patients, 3 (43%) dropped out before treatment began, and 4 (57%) were treated by means of an online, Internet-based intervention based on CBT (iCBT). The intervention was primarily aimed at psychoeducation, treatment of residual depressive symptoms, emotion regulation, and improved sleep. All patients had ongoing pharmacological treatment at recruitment and established contact with a psychiatrist. The duration of BP-II among the treated patients was between 6 and 31 years. A single-subject design was used and the results of the 4 participating patients were presented individually.

**Results:**

Initiating treatment was perceived as too demanding under current life circumstances for 3 patients who consequently dropped out during baseline assessment. Self-ratings using the Montgomery-Åsberg Depression Rating Scale—Self-rated (MADRS-S) showed symptom reduction in 3 (75%) of the 4 treated cases during iCBT. In the evaluation of the treatment, 2 patients reported that they perceived that the treatment had reduced symptoms a little, 1 that it had reduced symptoms very much, and 1 not at all. Treatment adherence (ie, module completion) was fairly high in 3 cases. In general, the modules were perceived as fairly helpful or very helpful by the patients. In one case, there was a reliable change—according to the Reliable Change Index—in self-rated symptoms of depression and perseverative thinking.

**Conclusions:**

The treatment seemed to have acceptable feasibility. The iCBT intervention could be an effective way to treat residual symptoms in some patients with bipolar disorder type II. This should be investigated in a larger study.

**Trial Registration:**

ClinicalTrials.gov NCT01742351; https://clinicaltrials.gov/ct2/show/NCT01742351 (Archived by WebCite at http://www.webcitation.org/6XnVpv4C3).

## Introduction

The lifetime prevalence of bipolar disorder type II (BP-II) was estimated at 0.4% in a large international study in 2011 [1]. Although the prevalence might be low, BP-II remains a challenge for society due to the low age of onset—estimated to be approximately 20 years of age [2]—the chronic course of the illness, and the patient’s need of lifelong outpatient care, often in combination with recurring hospitalization [3]. Bipolar disorder does not only lead to high levels of sick leave [4], but also to large role impairments in other areas of life, and around 20% of patients will attempt suicide [1]. Furthermore*,* comorbidity is high, especially with anxiety and substance abuse [1].

Residual symptoms are very common between the hypomanic or depressed episodes. On average, sufferers of BP-II can expect to experience symptoms more than half of the time [5], with continuing role impairment as a consequence in many cases [6]. Prospective studies [7,8] have revealed that bipolar patients with residual symptoms relapse considerably faster compared to those in full remission. Interventions targeting residual symptoms are clearly needed and psychological interventions, for example, cognitive behavioral therapy (CBT), have been used for this purpose [9]. Recent evidence shows that combining CBT with medication is effective in bipolar patients as it reduces symptoms [10] and lowers the risk of relapse [11]. However, while pharmacotherapy for bipolar disorder is widely available, access to CBT is often limited.

In the treatment of unipolar depression, there is a similar situation with limited access to effective psychological treatment. This has resulted in the emergence of Internet-based interventions for major depression based on cognitive behavioral therapy (iCBT), which has been shown to be effective in several studies, as well as in two recent meta-analyses [12,13].

Internet-based psychoeducation for bipolar patients—type I*,* type II, and not otherwise specified (NOS)—in full remission has been tested in a pilot study with a small beneficial effect on psychological quality of life compared to a control group, but with no effect on depressive symptoms during follow-up [14]. In a randomized controlled trial (RCT), no difference was found between Internet-based psychoeducation for bipolar disorder—any subtype—and a control condition on the reduction of depressive symptoms, although it was also demonstrated that guidance increased adherence to the online program compared to a completely self-guided intervention [15]. More trials on Internet-based interventions for bipolar disorder are being conducted [16-18], but few results have been published.

The aim of this study was to investigate the feasibility of iCBT and its effect on residual symptoms in persons diagnosed with BP-II. As the application of iCBT for bipolar disorder is a largely unexplored area, we employed a replicated single-case experimental design. While this design provides the opportunity to draw valid inferences of treatment effectiveness, the requirement to collect data on large and homogeneous groups is circumvented. Thus, the design is well suited to testing initial feasibility and obtaining a preliminary indication of effectiveness.

Specifically, this pilot study aims to answer the following questions:

Is iCBT a feasible approach? Specifically, (1) to what extent do patients show interest and participate in the treatment? and (2) to what extent do patients experience the treatment as helpful?

Does iCBT affect residual symptoms? Specifically, (1) does the intervention lead to a reduction in depressive symptoms? and (2) does the intervention lead to a reduction in sleep problems?

## Methods

### Participants and Procedure

The inclusion criteria were as follows: a minimum age of 18 years, a diagnosis of BP-II, stable and adequate pharmacological treatment for BP-II (ie, no medication change in the previous 3 months, antidepressants only allowed in the presence of mood stabilizers), Internet access, ability to read and write Swedish, and mild-to-moderate residual depressive symptoms defined as a score on the Montgomery-Åsberg Depression Rating Scale—Self-rated (MADRS-S) [19] of no less than 7 and no higher than 34 [20]. Exclusion criteria were as follows: having been diagnosed with a psychotic disorder or hospitalized within psychiatric care during the previous 12 months, previous suicide attempts, documented parasuicidal behavior or a score above 3 on item 9 on the MADRS-S—which would indicate suicidality—a history of mania, or ongoing psychotherapy.

The trial protocol was approved by the Regional Ethical Review Board in Uppsala (No. 2012/341) and registered at ClinicalTrials.gov (NCT01742351).

A total of 548 patients were extracted from a psychiatry database held by Region Örebro County based on having been diagnosed, at least once, with other bipolar affective disorder (F31.8)—the formal categorization of bipolar II in the International Classification of Diseases, 10^th^revision (ICD-10) [21]—bipolar affective disorder, currently in remission (F31.7), or bipolar affective disorder, current episode hypomanic (F31.0). After review of the patient records, 477 out of 548 (87.0%) were excluded, most often due to having also been diagnosed with bipolar disorder type I (BP-I) at some point, a documented manic episode, or lack of a clear record entry where a psychiatrist had diagnosed the patient with BP-II. Letters were sent to the remaining 71 patients out of 548 (13.0%) with information about the study, and 18 out of 71 (25%) expressed an interest for further assessment. During a telephone interview, the patients were asked about Internet access and whether their language skills were sufficient for participation, at which time they had an opportunity to raise questions about the project. Those who provided consent received instructions about filling out the MADRS-S on the study website by logging on with a username and password. Their current medication was also assessed by a psychiatrist (MH) to ensure that it was stable and adequate for BP-II. Of the 18 patients, 5 (28%) were excluded due to recent changes of medication and 1 (6%) due to an inadequately high dosage of benzodiazepine. Of the 18 patients, 7 (39%) fulfilled the criteria and were given instructions to start a 3-week baseline assessment. During the baseline assessment, 3 patients of the 7 (43%) withdrew from further participation. The reasons given for dropping out were as follows: lack of time, lack of energy, and prioritizing the care for an ill family member. The remaining 4 patients out of 7 (57%) started the intervention and their data is presented in this article. After baseline, the 6-week intervention period began during which the patients worked with the treatment modules. Patients could communicate with a personal therapist throughout the treatment via secure emails. Therapist contact was typically used for support and for clarifying interventions, as well as for feedback on homework. The therapists also prompted patients who were inactive. Table 1 shows the characteristics of the patients at the baseline assessment.

**Table 1:** Patient characteristics at baseline.

Patient number	Age in years	Gender	Medication	MADRS-S^a^	Duration of BD-II^b^in years
1	66	Female	valproate, olanzapine^c^, fluoxetine, zopiclone (prn^d^)	19	14
2	31	Female	aripiprazole, duloxetine, propiomazine (prn), zolpidem (prn), levothyroxine	11	6
3	49	Female	lithium sulfate, melatonin, levothyroxine, orlistat	12	31
4	49	Male	valproate, lamotrigine, olanzapine^c^, venlafaxine	8	8
5^e^	24	Female	lamotrigine, olanzapine^c^, sertraline	18	2
6^e^	32	Female	lamotrigine, fluoxetine, pregabalin, oxazepam (prn), zopiclone (prn), acetaminophen, pramipexole	20	3
7^e^	56	Female	lithium sulfate, propiomazine	10	30

^a^score on the Montgomery-Åsberg Depression Rating Scale—Self-rated (MADRS-S) at baseline screening

^b^years since first diagnosed with bipolar disorder type II (BD-II)

^c^The Olanzapine dosage for patient 1 was 5 mg hs (at bedtime), and for patients 4 and 5 was only 2.5 mg hs, thus hardly interfering with daytime cognition

^d^prn: as needed

^e^patients 5, 6, and 7 dropped out during baseline

### Design

**Figure 1:**
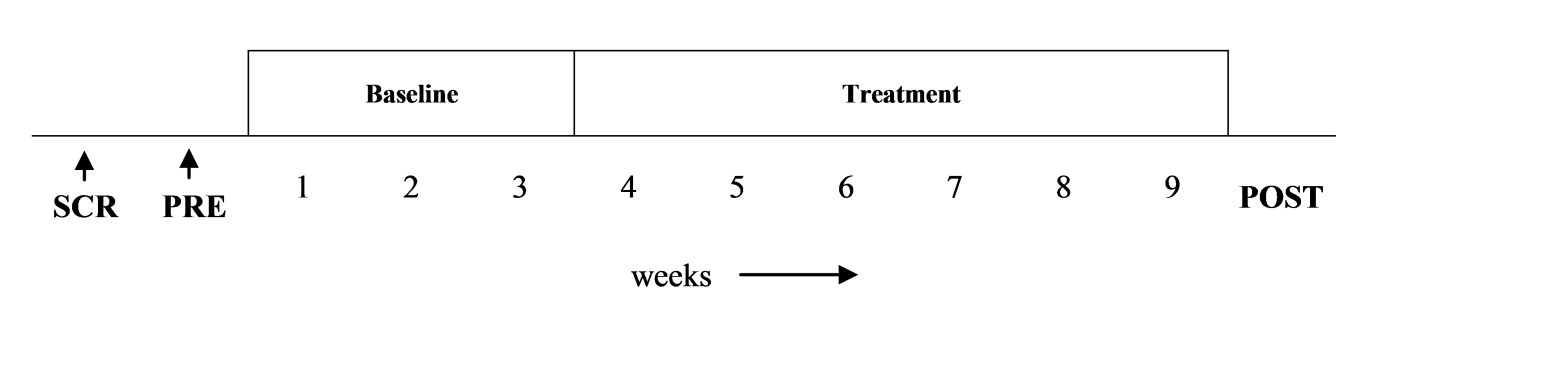
An overview of the study design. SCR: initial screening (MADRS-S), PRE: pretest (BDI-II, PTQ, WSAS), Baseline: baseline assessment with weekly ratings (MADRS-S, ISI), Treatment: intervention phase comprising assessment with weekly ratings (MADRS-S, ISI), POST: posttest (BDI-II, PTQ, WSAS, patient evaluation).

A replicated single-case experimental design was used with a 3-week baseline period and a 6-week treatment period. The 4 participants functioned as their own control group in this design and the primary analysis was a visual comparison of the scores during baseline and treatment (see Figure 1).

### Measures

The primary outcome (ie, depressive symptoms) was measured by an Internet-based version of the Montgomery-Åsberg Depression Rating Scale—Self-rated [19] on a weekly basis during the baseline period and the treatment phase. The MADRS-S is a 9-item self-report measure that generates a total score from 0 to 54, with higher scores indicating more severe depressive symptoms. It has good internal consistency*,* for instance, a Cronbach alpha of .84 [22]*,* and has been validated for online use [23]. Item 9 was employed to detect suicidality. When using this Internet-based version, the respondent sees 1 item per frame but can go back and change previous answers until the last question is answered. Only one alternative can be chosen per item and it is not possible to skip items.

The Beck Depression Inventory—Second Edition (BDI-II) [24] was employed to measure depressive symptoms before and after treatment. The BDI-II is a 21-item self-report measure that was used as a complement to the MADRS-S. The instrument’s psychometric properties have been shown to be very good [25] and it has been validated for online use [23].

The Insomnia Severity Index (ISI) [26] was employed weekly to measure the severity and impact of sleeping problems. It is a 7-item self-report measure with excellent internal consistency and generates a score between 0 and 28 [27].

The Work and Social Adjustment Scale (WSAS) [28] was employed to measure the level of functioning of the participating patients. This 5-item self-report measure was administered before and after treatment.

The Perseverative Thinking Questionnaire (PTQ) [29] is a 15-item self-report instrument that was used to assess repetitive negative thinking before and after treatment. The internal consistency has been shown to be excellent [29].

The Affective Self-Rating Scale (AS-18) for manic, depressive, and mixed states is an 18-item self-report measure with very good internal consistency [30]. It is divided into two subscales—depressive and (hypo)manic—and in this study we used the subscale that measures mania by means of 9 items with a score ranging from 0 to 36 in order to detect any deterioration into manic episodes.

Patient satisfaction was measured by the question “How satisfied are you with the treatment?” Answers were given on a 5-point scale from “Very dissatisfied” (1) to “Very satisfied” (5). The perceived helpfulness of the modules in the treatment was rated by the patients on a 5-point scale from “Unhelpful” (1) to “Very helpful” (5). The patient also rated if, and how much, their problems had decreased during treatment using a 5-point scale from “Not at all” (1) to “Very much” (5).

### Intervention and Therapist Contact

The iCBT material comprised six modules*,* one of which was to be completed every week for 6 weeks. The modules were (1) Psychoeducation, (2) Emotion regulation by behavioral activation and regularity in day-to-day life, (3) and (4) Improving sleep quality, (5) Cognitive restructuring, and (6) Long-term goals and relapse prevention. The modules contained theoretical information, treatment rationale, examples, work sheets, and homework assignments. At the end of each module there were questions about the theoretical content, as well as homework. The patients did not gain access to the next module until they had sent written responses to their therapist. The individual module should have been seen as a chance to learn about a topic relevant to BP-II, and a chance to try and evaluate new strategies. Patients were encouraged to spend time practicing the strategies they perceived as effective, and to incorporate them into daily life so that they could continue to benefit from them after the end of treatment. The total amount of text in the modules was slightly above 30,000 words. A secure system for asynchronous emails was used for the therapist contact. It was not restricted, for instance, the participants chose the frequency of the contact. The therapists were supervised by a clinical psychologist experienced in Internet treatment, and an effort was made to be clear about the framework early in the project, for example, what the patient could expect from the therapist. This and other features of the support were inspired by supportive accountability [31]. There was no face-to-face contact between patients and therapists.

### Analyses

In order to judge feasibility, the degree of interest in the study exhibited by eligible patients, as well as dropout rate, is described. In addition, the evaluation of the 4 participants who completed the intervention is presented. In the evaluation, patients were asked to rate satisfaction, perceived decrease of problems, and helpfulness of each module on a 5-point scale. To investigate the effectiveness of the intervention on symptoms, analyses of pre-/posttest differences in depression (BDI-II), repetitive negative thinking (PTQ), and function (WSAS) were conducted. The Reliable Change Index (RCI)—defined by Jacobson and Truax in 1991 [32]—was calculated to investigate whether there was a reliable difference between depression, repetitive thinking, and function scores before and after the intervention period. For this calculation, the standard deviation and test/retest reliability of the BDI-II, the PTQ, and the WSAS were obtained from previous research [28,29,33]. The weekly scores measuring depressive symptoms (MADRS-S), sleep problems (ISI), and symptoms of (hypo)mania (AS-18) across baseline (3 weeks) and treatment (6 weeks) were graphically displayed, and visual analyses conducted, in order to detect differences in levels and trends between baseline and treatment [34]. For the primary outcome (MADRS-S) the mean of each phase—baseline and treatment—was calculated and marked on the graph as a dashed horizontal line.

## Results

### Treatment Feasibility

#### To What Extent Do Patients Show Interest and Participate in the Treatment?

As described under Participants and Procedure in the Methods section, 71 patients were invited to participate in the study based on initial screening of patient records. Of these*,* 18 (25%) responded and exhibited an interest in participating in the treatment. Of the 18 patients, 3 (17%) subsequently withdrew from the study and 8 (44%) were excluded with reference to the predefined exclusion criteria. Of the 18 patients, 7 (39%) were thus offered participation in the study, of whom 3 (43%) dropped out during baseline assessment or before the start of the intervention. Of the 7 patients, 4 (57%) started the treatment and all of them participated during the full intervention period of 6 weeks. Patients 1 and 3 completed all six modules. Patient 4 completed four of the modules and started the fifth. Patient 2 completed the first module and started the second.

#### To What Extent Did the Participants Experience the Treatment as Helpful?

The 4 patients who participated filled out a treatment satisfaction evaluation form at the end of the treatment period. Table 2 presents the results of their evaluations.

As can be seen in Table 2, patient 1 was very satisfied with the treatment, patient 4 was fairly satisfied, and patients 2 and 3 were neither satisfied nor dissatisfied. Patient 1 stated that her problem decreased very much as a result of the treatment. Patients 3 and 4 reported that their problems decreased a little, while patient 2 stated that her problems did not decrease at all. Patient 1 experienced all modules as very helpful. Patient 4 perceived modules 3 and 4—sleep modules—as very helpful and the other modules as fairly helpful. Patient 3 experienced modules 3 and 4 as fairly helpful and the other modules as neither helpful nor unhelpful, or fairly unhelpful. Patient 2 experienced modules 1 and 2 as fairly helpful—the only modules that patient 2 received.

In summary, 25% (18/71) of patients who were invited to participate in the study reported interest. The participants who completed more than four modules felt that their problems decreased a little, or largely, as a result of the treatment. All participants found that some or all of the modules were fairly, or very, helpful.

### Treatment Effect

#### Can Any Symptom Change Be Observed in the Weekly Self-Reports?

The weekly ratings of depressive symptoms (MADRS-S), symptoms of (hypo)mania (AS-18), and insomnia (ISI) for the 4 patients are discussed in the following sections, and illustrated in the following Figures.

#### Patient 1

**Figure 2:**
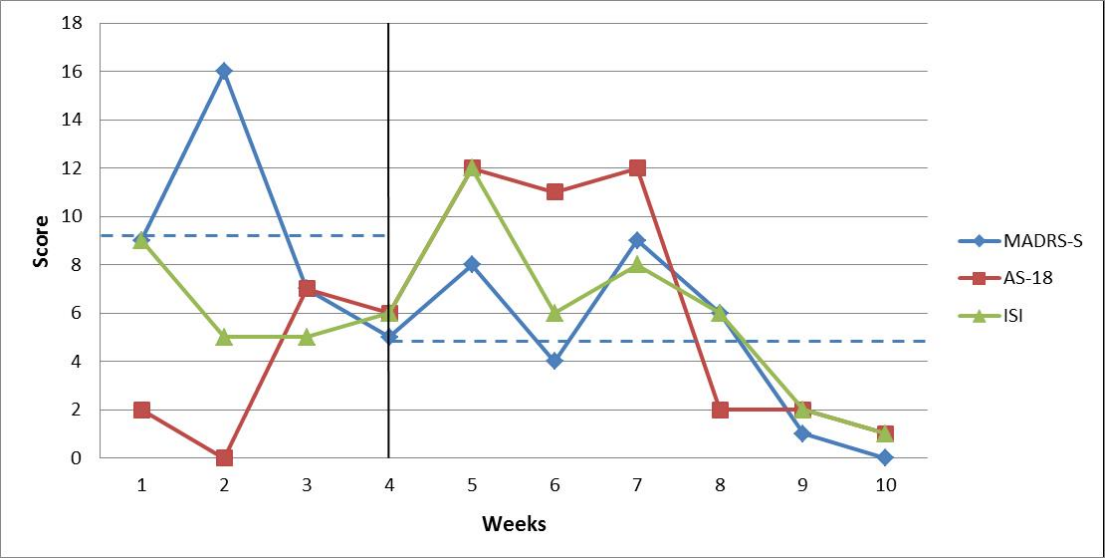
Weekly ratings of depressive symptoms (MADRS-S, maximum score is 54 points), symptoms of (hypo)mania (AS-18, maximum score for mania section is 36), and insomnia (ISI, maximum score is 28 points) for patient 1. The vertical line marks the start of the intervention period. Dashed blue lines indicate the mean level of depressive symptoms (MADRS-S) during the baseline period and the treatment phase.

The weekly MADRS-S ratings showed that patient 1 had an average of 9.25 (SD 4.79) points during the 3-week baseline period and 4.7 (SD 3.7) points during treatment. During the baseline period, the scores were in a subclinical range on three of the four occasions and in the range of mild depression—value of 16—on one occasion. During treatment, all scores were at a subclinical level and the last two measurement occasions revealed a very low presence to complete absence of depressive symptoms. Both phase averages were within the range of subclinical depressive symptoms. Visually, a difference in level between the phases could be inferred. The change was gradual, indicating a long latency. For patient 1, the averages of the weekly sleep problem scores were the same—6.3 points—during the baseline period (SD 2.3) and the treatment phase (SD 4.0). The visual analysis showed a stable baseline with scores within the subclinical range. A slight increase in perceived sleep problems occurred in connection with the first week of treatment and then decreased continuously during subsequent weeks. Symptoms of hypomania were for the most part in the subclinical range, but on two occasions in the area a hypomanic episode might have been suspected for patient 1, but the symptoms soon returned to a subclinical level. Figure 2 shows the weekly estimates by patient 1 for all symptoms.

#### Patient 2

**Figure 3:**
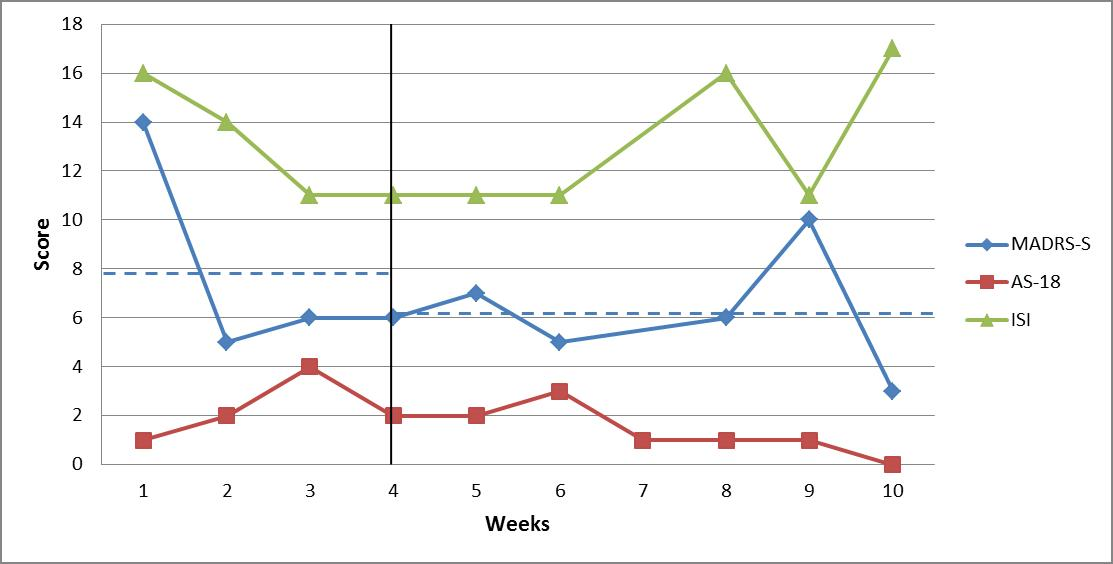
Weekly ratings of depressive symptoms (MADRS-S, maximum score is 54 points), symptoms of (hypo)mania (AS-18, maximum score for mania section is 36), and insomnia (ISI, maximum score is 28 points) for patient 2. The vertical line marks the start of the intervention period. Dashed blue lines indicate the mean level of depressive symptoms (MADRS-S) during the baseline period and the treatment phase.

For patient 2, the average of the weekly scores of depressive symptoms (MADRS-S) was 7.75 (SD 4.20) points during the baseline period and 6.2 (SD 2.6) points during treatment. The baseline scores were in a subclinical range on three of the four occasions, and in the range of mild depression on one occasion—value of 14. During the treatment phase, all ratings of depressive symptoms ​​were on a subclinical level. For patient 2, the averages of the weekly insomnia symptom scores were the same—13 points—during both the baseline period (SD 2.4) and the treatment phase (SD 3.0). During baseline, the scores were within the subclinical range, except for the first measurement where the value indicated clinically significant insomnia (ie, intermediate). During treatment, the scores varied between subclinical and clinically significant insomnia (ie, intermediate). The AS-18—symptoms of hypomania—scores were low during the whole study for patient 2, indicating no problems with hypomania or mania. Figure 3 shows the weekly estimates by patient 2 for all symptoms.

**Table 2:** Results from the patient evaluation form distributed after completion of the intervention period.

Evaluation categories and responses		Patients
**Treatment satisfaction**		
	Very dissatisfied	
	Fairly dissatisfied	
	Neither satisfied nor dissatisfied	Patient 2, Patient 3
	Fairly satisfied	Patient 4
	Very satisfied	Patient 1
**Perceived problem decrease**		
	Not at all	Patient 2
	A little	Patient 3, Patient 4
	Some	
	A lot	
	Very much	Patient 1
**Perceived helpfulness of module 1** ^**a,b**^		
	Unhelpful	
	Fairly unhelpful	
	Neither helpful nor unhelpful	Patient 3
	Fairly helpful	Patient 2, Patient 4
	Very helpful	Patient 1
**Perceived helpfulness of module 2**		
	Unhelpful	
	Fairly unhelpful	
	Neither helpful nor unhelpful	Patient 3
	Fairly helpful	Patient 2, Patient 4
	Very helpful	Patient 1
**Perceived helpfulness of module 3**		
	Unhelpful	
	Fairly unhelpful	
	Neither helpful nor unhelpful	
	Fairly helpful	Patient 3
	Very helpful	Patient 1, Patient 4
**Perceived helpfulness of module 4**		
	Unhelpful	
	Fairly unhelpful	
	Neither helpful nor unhelpful	
	Fairly helpful	Patient 3
	Very helpful	Patient 1, Patient 4
**Perceived helpfulness of module 5**		
	Unhelpful	
	Fairly unhelpful	Patient 3
	Neither helpful nor unhelpful	
	Fairly helpful	Patient 4
	Very helpful	Patient 1
**Perceived helpfulness of module 6**		
	Unhelpful	
	Fairly unhelpful	
	Neither helpful nor unhelpful	Patient 3
	Fairly helpful	
	Very helpful	Patient 1

^a^Patient 2 only received modules 1 and 2.

^b^Patient 4 only received modules 1 to 5.

#### Patient 3

**Figure 4:**
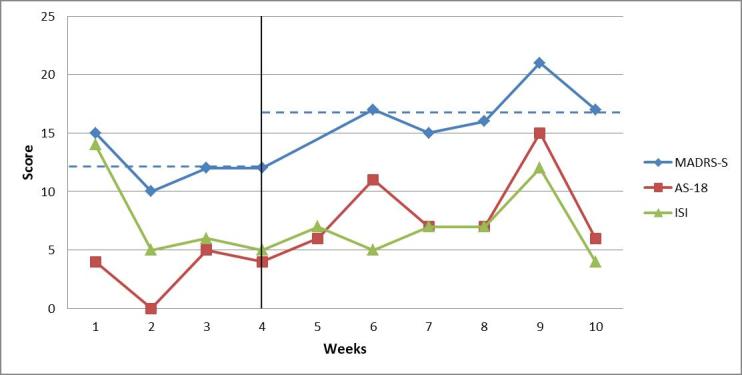
Weekly ratings of depressive symptoms (MADRS-S, maximum score is 54 points), symptoms of (hypo)mania (AS-18, maximum score for mania section is 36), and insomnia (ISI, maximum score is 28 points) for patient 3. The vertical line marks the start of the intervention period. Dashed blue lines indicate the mean level of depressive symptoms (MADRS-S) during the baseline period and the treatment phase.

For patient 3, the average of the weekly ratings of depressive symptoms was 12.25 (SD 2.10) points during the baseline period and 17.2 (SD 2.3) points during treatment. Visually, there was a change between phases*,* suggesting a higher level of depressive symptoms during treatment. During the baseline period, the scores were in the mild depression range on three of the four occasions and on a subclinical level on one occasion. During treatment, most values ​​were within the mild depression range, but one value—value of 21—reached the limit of moderate depression. For patient 3, the averages of the weekly ratings of insomnia symptoms were almost identical—7.5 (SD 4.4) during the baseline period and 7.0 (SD 2.8) during treatment. These ratings were within the subclinical range during both the baseline and the treatment phase. The hypomania symptom ratings showed an increasing trend during the baseline period and the treatment phase, and at one point they were clinically significant, but returned quickly to a subclinical level. Figure 4 shows the weekly estimates by patient 3 for all symptoms.

#### Patient 4

**Figure 5:**
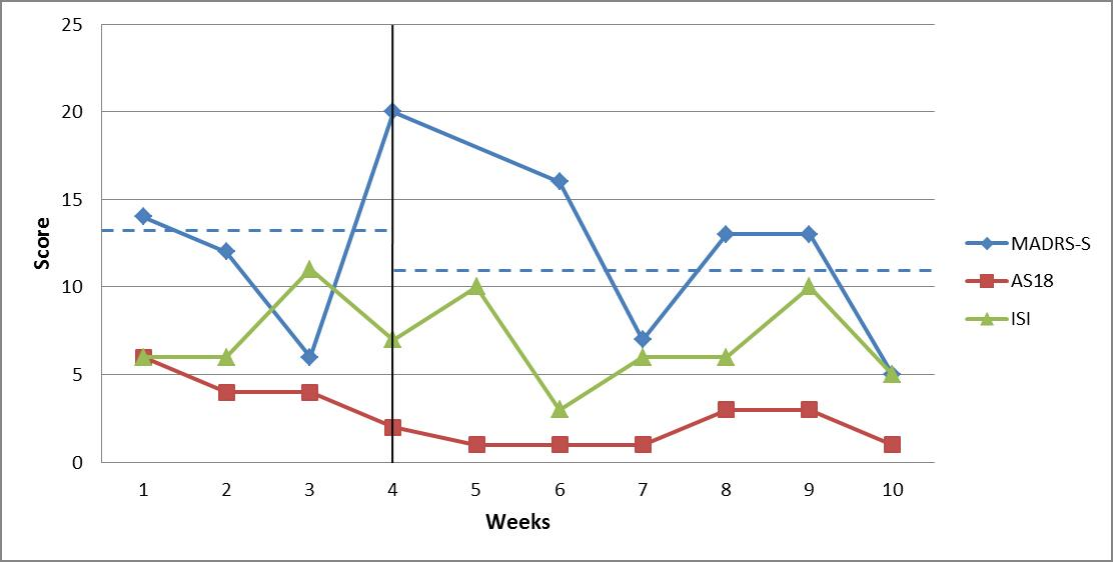
Weekly ratings of depressive symptoms (MADRS-S, maximum score is 54 points), symptoms of (hypo)mania (AS-18, maximum score for mania section is 36), and insomnia (ISI, maximum score is 28 points) for patient 4. The vertical line marks the start of the intervention period. Dashed blue lines indicate the mean level of depressive symptoms (MADRS-S) during the baseline period and the treatment phase.

For patient 4, the average of the weekly ratings of depressive symptoms was 13.0 (SD 5.8) points during the baseline period and 10.8 (SD 4.6) points during treatment. The visual analysis showed a relatively high degree of fluctuation in both phases, making it difficult to draw conclusions regarding a change in level. During the baseline period, the ratings fluctuated between subclinical symptoms, mild depression*,* and moderate depression. During the treatment phase, the ratings varied between subclinical symptoms and mild depressive symptoms. For patient 4, the average of the weekly insomnia ratings (ISI) was 7.5 (SD 2.4) points during the baseline period and 6.7 (SD 2.8) during the treatment. The AS-18—symptoms of hypomania—ratings were low during the whole study for patient 4, indicating no problem with hypomania or mania. Figure 5 shows the weekly estimates by patient 4 for all symptoms.

#### Pre-/Posttest Evaluations for All Patients

Table 3 presents the patients’ pre-/posttest ratings of depressive symptoms (BDI-II), repetitive negative thinking (PTQ), and function (WSAS). Between the pre- and posttest ratings, patient 1 exhibited a reliable change on the BDI-II and the PTQ, while patients 2, 3, and 4 did not exhibit any significant changes between pre- and posttest ratings.

**Table 3:** Pre-/posttest evaluation on BDI-II, PTQ, and WSAS.

Patient number	Evaluation of symptoms											
	Depression (BDI-II)^a^				Repetitive thinking (PTQ)^b^				Function (WSAS)^c^			
	Pre^d^	Post^d^	RCI^e^	*P*	Pre	Post	RCI	*P*	Pre	Post	RCI	*P*
1	12	0	- 2.52	*.01* ^f^	33	0	- 4.20	<*.001*	20	24	0.35	.73
2	10	1	- 1.89	.06	24	13	- 1.40	.16	14	14	0	1
3	20	25	1.05	.29	10	10	0	1	15	26	0.97	.33
4	9	6	- 0.63	.53	39	34	- .64	.52	11	6	- 0.44	.66

^a^As compared to bipolar patients (n=34) as reported in Beck et al [33], Beck Depression Inventory—Second Edition (BDI-II).

^b^As compared to depressed patients (n=45) as reported in Ehring et al [29], Perseverative Thinking Questionnaire (PTQ).

^c^As compared to mild to moderately depressed patients (n=382) as reported in Mundt et al [28], Work and Social Adjustment Scale (WSAS).

^d^Pretest value (Pre), posttest value (Post)

^e^Reliable Change Index (RCI)

^f^Significant values are shown in italics (*P*<.05).

## Discussion

### Principal Findings

The results of this study indicate that for some individuals with BP-II, iCBT can lead to a decrease in depressive symptoms. Our results also revealed that there is an interest in Internet-based CBT on the part of such patients. Module completion was fairly high in 3 out of 4 cases, and the patients mostly rated the treatment modules as fairly or very helpful. On the whole, the patients who initiated iCBT were satisfied with, or neutral toward, the treatment. Of the patients, 1 reported that her problems did not decrease at all, but she only completed one module. Of the patients, 2 felt that their problems had decreased a little and 1 felt that the problems had decreased very much*.* Entering treatment was perceived as too demanding under current life circumstances by the 3 patients who dropped out during the baseline assessment. As there was interest in the treatment, and module completion was fairly high, we deem the feasibility of the treatment to be acceptable.

CBT has previously had a favorable outcome among bipolar patients when delivered face-to-face [35]. This study indicates that the intervention can also be effectively administered via the Internet. However, the results should be replicated in larger studies, and if found to be efficacious it could improve access to psychological treatment for many patients with a serious mental condition. We are not aware of any previous research on iCBT for BP-II, thus this study clearly adds to the knowledge about Internet-based CBT and future possibilities for treating the disorder. A study on Internet treatment of bipolar disorder type I and type II has been published recently with positive results [36]. Lauder et al found that 48% of their participants completed all the modules, which is similar to the adherence in this study.

There were some seemingly contradictory results in our study in that patients were fairly satisfied with the intervention and considered the modules helpful, but at the same time 3 out of 4 individuals perceived little or no reduction of their problems. One explanation could be that symptom reduction is not the only outcome desired by patients*,* which is in line with the findings from a qualitative study on an Internet-based intervention for bipolar disorder [37]. The researchers found that learning to live with bipolar disorder (ie, experience, knowledge, and skills) was just as important for some participants as reduced symptoms. The participants actually considered these two goals intertwined in creating a good quality of life.

Somewhat surprisingly, there was almost no effect on sleep problems during treatment in the 4 patients, which is in contrast to an earlier study demonstrating the effect of iCBT on insomnia [38]. However, the patients in this study suffered from BP-II and, thus, had a more severe psychopathology. In addition, their levels of sleep disturbance were rather low from the outset in most cases, and the treatment duration was only 6 weeks. Perhaps more comprehensive treatment material about sleep is necessary to improve the effect.

By communicating with the patients, we gained the impression that the treatment may need some alterations. First, we think that its duration should be longer to allow the patient more time to perform the exercises in the modules. This would also be more in line with other treatments for unipolar depression and anxiety*,* which often comprise 10 to 12 weeks [39,40]. There are also indications that it takes time to establish and maintain behavior change in patients with bipolar disorder [41]. A module about pharmacological treatment and side effects could have been included. Such a module was pilot-tested during development of an Internet-based psychoeducational intervention for bipolar disorder by Latalova et al and received a great deal of interest from patients [41]. Perhaps the modules about sleep would have had a greater impact if the therapists had monitored the ISI ratings more closely and used them actively in their feedback to the patients. A module could also be included that systematically aims to involve a next of kin in the treatment.

### Limitations

A few limitations should be acknowledged. First, as there was no follow-up we were unable to obtain knowledge of symptom levels during the time period after the treatment. Second, we included few patients and some dropped out, therefore, our results cannot be generalized to all individuals who suffer from BP-II. Third, we relied on diagnoses from patient records instead of diagnosing the patients ourselves. This makes it likely that not all of them were diagnosed using the same (ie, standardized) procedure, although on the other hand the cases are likely to be representative in severity of patients with BP-II in a clinical setting. A strength of the study is, therefore, ecological validity as well as the fact that we used patient-rated outcome of depressive symptoms. The risk of an allegiance effect is reduced compared to clinician-rated outcome. Another strength is that the self-report measures of depressive symptoms have been validated for online use. However, complementary objective measures could have increased reliability.

More studies on this treatment are necessary. An experimental design could be used to assess the relative efficacy of the Internet-based intervention by comparing it with an established form of therapy (eg, group CBT). A study with a larger sample could possibly include patients with BP-I and BP-II. A longer follow-up time is needed and relapse, as well as hospitalization, should be included as variables in future studies.

### Conclusions

This small pilot study showed that iCBT can have an effect on depressive symptoms in some patients with BP-II. This should be further investigated in larger studies.

## Abbreviations

AS-18: Affective Self-Rating Scale
BDI-II: Beck Depression Inventory—Second Edition
BP-I: bipolar disorder type I
BP-II: bipolar disorder type II
CBT: cognitive behavioral therapy
hs: at bedtime
iCBT: Internet-based cognitive behavioral therapy
ICD-10: International Classification of Diseases, 10threvision
ISI: Insomnia Severity Index
MADRS-S: Montgomery-Åsberg Depression Rating Scale—Self-rated
NOS: not otherwise specified
prn: as needed
PTQ: Perseverative Thinking Questionnaire
RCI: Reliable Change Index
RCT: randomized controlled trial
WSAS: Work and Social Adjustment Scale
